# FORWARD STEPS AND MISSTEPS: WHAT WE’VE LEARNED THROUGH THE PROCESS OF COMMUNITY-ENGAGED RESEARCH

**DOI:** 10.1093/geroni/igad104.0279

**Published:** 2023-12-21

**Authors:** Jordan Lewis

**Affiliations:** University of Minnesota Medical School, Duluth campus, Duluth, Minnesota, United States

## Abstract

Historically, research with Indigenous populations has disregarded community input, creating mistrust among tribal communities toward researchers, and resulting in communities limiting their involvement in research projects. Today, Indigenous communities are becoming more engaged in the research process, including developing their own tribal review boards, partnering with researchers throughout entire research process, as well as exercising their tribal sovereignty and housing their own data. These efforts have resulted in the development of rigorous Indigenous approval processes, increased engagement of Indigenous people and communities in research, and studies that have direct impacts on those engaged, as well as future generations, as well as protect both the Indigenous communities and the researchers. As a researcher trained to conduct culturally respectful and culturally safe research with Indigenous communities in Alaska, my team and I have gained first-hand experience with tribal research protocols, research processes, and the iterative process of developing relationships, navigating research ethics, as well as ensuring that findings are reflective of the community, its individual members, and the region. Despite our best efforts, research does not go as written in our grant proposals. This presentation will outline the complexities of navigating tribal approval processes in Alaska, highlight some of the challenges we have faced while conducting community-engaged research in Alaska; our lessons learned by doing it incorrectly. We will also share recommendations for researchers interested in working with Indigenous communities and how to work collaboratively with to avoid pitfalls, but also how to get back on track and develop lasting relationships.

